# Cancer Patients Circadian Rhythm Assessment Based on Morningness‐Eveningness Preference: A Cross‐Sectional Study

**DOI:** 10.1002/hsr2.71210

**Published:** 2025-09-03

**Authors:** Ghazal Daftari, Mahsa Abbaszadeh, Sahar Karimpour Reyhan, Nasim Khajavirad

**Affiliations:** ^1^ Department of Internal Medicine Imam Khomeini Hospital Complex Tehran University of Medical Sciences Tehran Iran; ^2^ Endocrinology and Metabolism Research Center (EMRC), Vali‐Asr Hospital, Imam Khomeini Hospital Complex Tehran University of Medical Sciences Tehran Iran

**Keywords:** cancer, chronotype, circadian rhythm, clock genes, MEQ

## Abstract

**Background and Aim:**

The circadian rhythm regulates various physiological processes, including sleep‐wake cycle, cell division and cancer development. This study aimed to investigate circadian rhythm patterns in cancer patients.

**Methods:**

In this cross‐sectional study, 150 cancer patients admitted to the hospital enrolled the study during the fall of 2022. The demographic characteristics of patients were collected using a checklist. Patients also completed a morningness‐eveningness questionnaire (MEQ). Utilizing Analysis of Variance (ANOVA) and Fisher's exact test, circadian rhythm types with continuous and categorical variables were compared.

**Results:**

The mean age of the study′s participants was 49.83 ± 14.53 years. A total of 82.7% (*n* = 124) had non‐hematological cancers, and breast cancer was the most prevalent type of cancer among patients (23.3%). The MEQ of the patients studied ranged from 41 to 74, with a mean score of 56.6 ± 6.34.

**Conclusion:**

According to the findings of this study, the circadian rhythm is distributed normally among the participants.

## 1 Introduction

The circadian clock with a 24‐h period is an essential biological and behavioral system that persists in plants, animals, and humans and regulates a variety of processes, including the cell cycle, body temperature, immunity, heartbeat, basal metabolism, blood pressure, hormone secretion, sleep‐wake cycle, feeding, and glucose homeostasis [1]. The system is coordinated with environmental stimuli such as food intake, light, and temperature [2]. In humans, the circadian rhythm consists of two components: the brain clock in the anterior hypothalamus, supra chiasmatic nucleus (SCN), and the peripheral clock in the individual cells [3, 4, 5, 6]. The brain clock regulates the peripheral clock of cells and functions as a pacemaker [3, 4, 5, 6].

Cellular clocks have autonomous rhythms and answer to intermittent signals from the SCN [3, 4, 5, 6].

Additionally, circadian pathway genes control the circadian rhythm [7, 8]. In 1917, 1978, 1997, and 2001, the clock genes were reported for the first time in the fruit fly *Drosophila*, the fungus *Neurospora*, the rat, and finally the human, respectively [9]. In 2017, the Nobel prize was awarded to scientists who discovered critical molecular mechanisms that control the circadian rhythm [10, 11]. The circadian rhythm is regulated by clock genes, as extensively reviewed in studies on the molecular architecture of the circadian clock [12].

Cancer is a significant cause of death worldwide [13]. Cancer incidence has increased due to aging, changes in associated risk factors, and socioeconomic developments [14]. Furthermore, some mutations in DNA replication and exogenous or endogenous DNA damage are associated with cancer [15, 16]. Several studies showed that disruption in circadian rhythm results in metabolic syndrome, endocrine disorders, and cancer [17]. In circadian patterns, individuals are typically classified as morning or evening types [18]. Morning types wake up early and function better in the morning [18]. Evening types stay up late and perform best at night [18]. Dysregulation of circadian rhythms in humans is associated with different cancers such as hepatocellular carcinoma [19], colon [20], ovarian cancers [21], prostate [22], and lung [23]. Consequently, the proper function of clock proteins and circadian rhythm plays a crucial role in cancer prevention and treatment [24]. Recent studies indicate bidirectional regulation between circadian genes and tumor signaling pathways, implicating circadian disruption in cancer pathogenesis. Considering the increasing recognition of circadian rhythm disruption in cancer development, progression, and response to treatment, the aim of this study was to evaluate chronotype distribution in cancer patients using the Morningness‐Eveningness Questionnaire (MEQ), with the goal of identifying potential implications for chrono‐therapeutic strategies. Since some patients were in the cachectic phase, this study also provides insight into chronotype patterns among individuals with advanced cancer.

## 2 Materials and Methods

### 2.1 Study Participants

This cross‐sectional study was conducted on 150 patients with hematologic and non‐hematologic cancers, ages 16–80, admitted to the internal medicine ward of Imam Khomeini Hospital Complex affiliated by Tehran University of Medical Sciences in the fall of 2022.

Initial estimates placed the sample size at 145 patients, based on a study indicating that circadian rhythm

disturbances and sleep disorders were prevalent in 30%–75% of cancer patients, or nearly twice the rate in the general population [25]. The sample size was calculated using the standard formula for estimating a single population proportion:

$$n\;=\;\left(Z^{2}\times p\times \left(1–p\right)\right)/d^{2}$$

where:

- **Z** = 1.96 (standard score for 95% confidence level)
- ***p*** = 0.05 (based on prior studies estimating circadian rhythm disruption in approximately 5% of cancer patients)
- **d** = 0.018 (margin of error)

An additional five participants were included to account for potential attrition or data exclusion. Inclusion criteria were age 15 or older, a cancer diagnosis, consent to participate in the study, absence of comorbidities, and absence of intubation. Exclusion criteria were using Melatonin, sedative, and hypnotic medications, significant cognitive impairment, night‐shift workers, and end‐stage patients. This study was conducted per the Declaration of Helsinki and was approved by the Imam Khomeini Hospital Complex, Tehran University of Medical Science, Tehran, Iran, Ethics Committee (ethics number: IR.TUMS.IKHC.1398.28). All patients were informed of the study, and consent forms were obtained.

### 2.2 Clinical and Demographic Evaluation

Researchers designed a questionnaire to collect demographic and clinical information, including gender, age, weight, height, marital status, education, occupation, type of cancer, duration of illness, number of hospitalizations, and history of chemotherapy or/and radiotherapy or/and surgery. The questionnaire's content was evaluated by specialists and deemed suitable for use (Appendix A).

### 2.3 The Morningness‐Eveningness Questionnaire

The circadian type of participants was evaluated using MEQ, which was initially presented by Ostberg and ¨ Horne [26]. MEQ is a self‐assessment questionnaire comprising 19 questions; the scores range from 16 to 86. The score weighting of the questions is not equal. This questionnaire describes five behavioral types: definitely morning type (70–86 scores), moderately morning type (59–69 scores), neither type (42–58 scores), moderately evening type (31–41 scores), and definitely evening type (16–30 scores) [27]. The Persian form of MEQ's validity and reliability have been established (*a* = 0.79) [28] MEQ data were collected once for each patient during this study period, regardless of their treatment phase (before, during, or after treatment).

A subset of participants was in the cachectic phase, which may influence circadian rhythms and other physiological parameters.

### 2.4 Statistical Analysis

Data were analyzed using SPSS software version 18.0 (SPSS Inc., Chicago, IL, USA). Descriptive statistics were used to summarize demographic and clinical characteristics. Continuous variables were presented as mean ± standard deviation (SD) or median and interquartile range (IQR) based on normality of distribution, which was assessed using the Kolmogorov–Smirnov analysis.

Categorical variables were reported as frequencies and percentages (n/N). Comparative analyses between circadian rhythm groups were conducted using ANOVA for continuous variables and Fisher's exact test for categorical variables.

All tests were two‐sided, and statistical significance was set at *p* < 0.05. Confidence intervals (95% CI) were reported where applicable.

## 3 Results

The age of patients ranged from 16 to 80 years, with a mean of 49.83 ± 14.53 years. BMI ranged from 12.41 to 41.79 kg/m², with a mean of 25.09 ± 5.32 kg/m². Table 1 and Table 2 show the participants' demographic, clinical, and medical characteristics.

**Table 1:** Demographic variables of study participants.

Variables	N	%
Gender		
Male	46/150	30.7
Female	104/150	69.3
Occupation		
Employed	52/150	34.7
Unemployed	98/150	65.3
Education		
Illiterate	24/150	16
Primary school	57/150	38
High school	53/150	35.3
Bachelor's degree	11/150	7.3
Master's degree	4/150	2.7
Ph.D. degree	1/150	0.7
Marital status		
Single	16/150	10.7
Married	134/150	89.3

**Table 2:** Clinical and medical variables of study participants.

Variables	N	%
Cancer type		
Breast	35/150	23.3
Colon	22/150	14.7
Ovary	16/150	10.7
Lung	7/150	4.7
Sarcoma	16/150	10.7
Gastric	11/150	7.3
Esophagus	4/150	2.7
Pancreas	1/150	0.7
Prostate	6/150	4
AML	8/150	5.3
ALL	6/150	4
Lymphoma	14/150	9.3
SCC	4/150	2.7
Chemotherapy		
Yes/No	137/13	91.3/8.7
Radiotherapy		
Yes/No	37/113	24.7/75.3
Surgery		
Yes/No	88/62	58.7/41.3
Patient condition		
Under treatment	83/150	55.3
Palliative therapy	67/150	44.7
Treatment		
Chemotherapy + Radiotherapy	32/150	21.33
Chemotherapy + surgery	80/150	53.33
Radiotherapy + surgery	28/150	18.66
Radiotherapy + surgery + chemotherapy	23/150	15.33

*Note:* Data are presented as frequency and percentage.

Among the participants, 17.3% (26/150) had hematologic cancers. Cancer incidence ranged from 1 to 180 months (23.26 ± 29.3). Among hospitalized patients, the number of chemotherapy sessions ranged from 1 to 13 (mean ± SD: 3.61 ± 2.37), radiotherapy sessions ranged from 0 to 70 (8.24 ± 9.79), and hospitalizations due to cancer ranged from 0 to 50 (5.50 ± 11.5). In this study, no cases of recurrence or relapse were reported during the data collection period.

**Figure 1:**
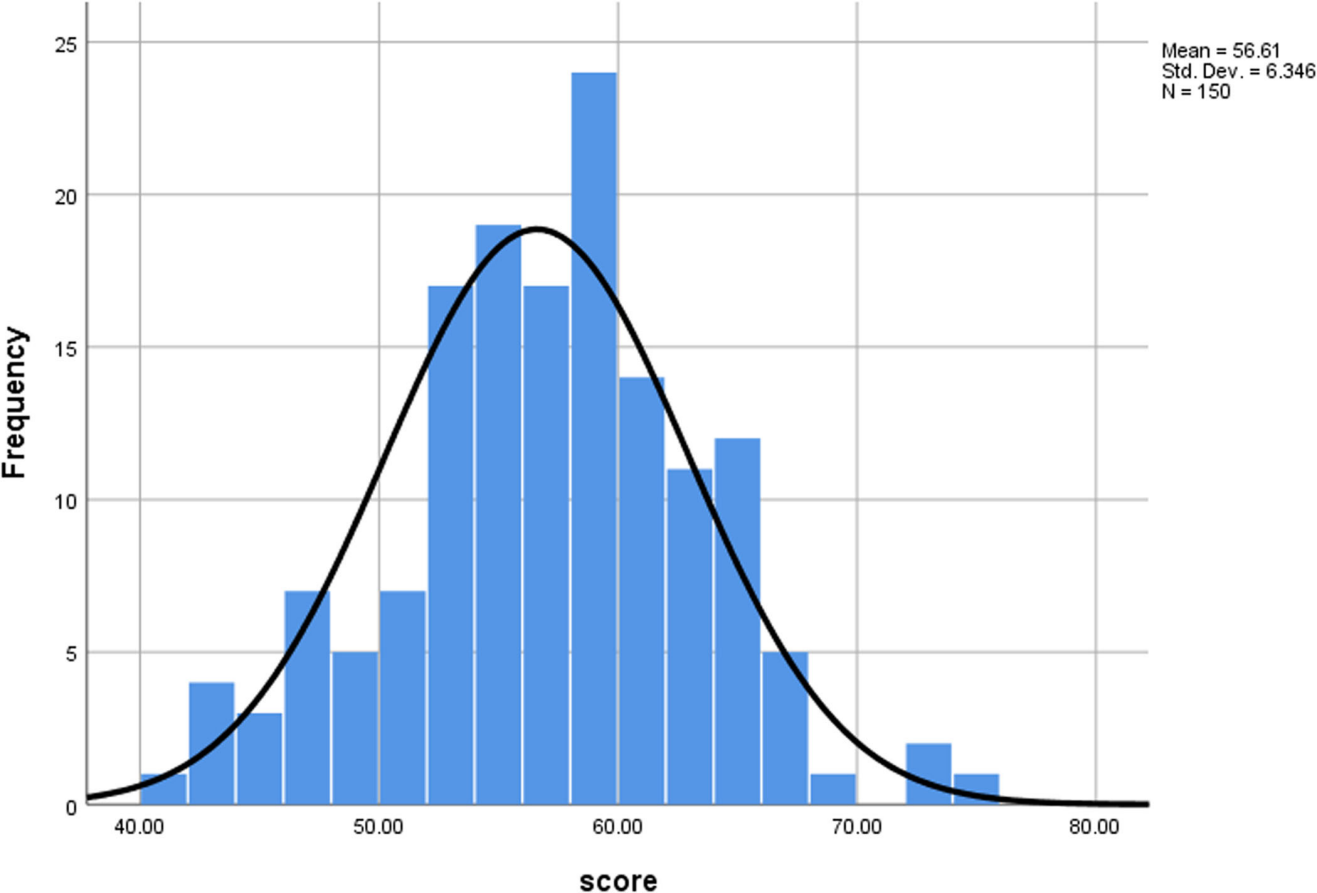
Distribution of chronotype categories based on MEQ scores among 150 cancer patients. The figure illustrates the relative proportions of morning, evening, and intermediate chronotypes.

Total MEQ scores ranged between 41 and 74 (56.6 ± 6.34). In this study, 2% (3/150) of patients were

definitely morning types, 38% (57/150) were moderately morning types, 59.3% (89/150) were neither type, 0.7% (1/150) was moderately evening types, and no patients were definitely evening types. Normal distribution was observed for MEQ scores among the participants (Figure 1).

According to Table 3, the mean ages of completely and moderately morning types were greater than those of all other types. The distribution of circadian rhythms was nearly identical between male and female participants. Employed and unemployed individuals, as well as patients with varying levels of education, were frequently morning and neither type. One‐way ANOVA showed no significant differences in age, time of cancer onset, number of hospitalizations, chemotherapy sessions, or radiotherapy sessions among different chronotype groups (*p* > 0.05 for all comparisons). Furthermore, Fisher's exact test found no statistically significant association between circadian rhythm type and categorical variables such as employment status, marital status, gender, type of treatment, education level, or clinical condition (*p* > 0.05 for all).

**Table 3:** Comparative analysis of demographic and clinical characteristics of patients with distinct circadian rhythm types.

Variable	Definitely morning type	Moderately morning type	Neither type	Moderately evening type	*p* value
Age (y)					
Mean ± S.D	56 ± 20.80	52.7 ± 12.52	47.97 ± 15.32	33	0.14¹
(Range)	(43–80)	(28–75)	(16–80)		
BMI (kg/m²)					
Mean ± S.D	29.84 ± 1.97	25.57 ± 5.74	24.62 ± 5.08	24.74	0.32¹
(Range)	(28.13–32.1)	(13.15–41.79)	(12.41–36.98)		
Cancer incidence (months)					
Mean ± S.D	16.33 ± 17.03	25.57 ± 5.74	24.62 ± 5.08	6	0.86¹
(Range)	(6–36)	(1–180)	(1–132)		
Hospital admissions					
Mean ± S.D	2.33 ± 1.15	3.21 ± 2.32	3.89 ± 2.42	5	0.25¹
(Range)	(1–3)	(1–13)	(1–10)		
Chemotherapy sessions					
Mean ± S.D	18.66 ± 17.00	7.96 ± 10.11	8.11 ± 9.31	4	0.3¹
(Range)	(6–38)	(0–48)	(0–70)		
Radiotherapy sessions					
Mean ± S.D	5.66 ± 9.81	4.05 ± 11.11	6.69 ± 11.84	0	0.56¹
(Range)	(0–17)	(0–50)	(0–40)		
Gender					
Male [*n* (%)]	1 (2.17)	21 (45.65)	24 (52.17)	0	0.63²
Female [*n* (%)]	2 (1.92)	36 (34.61)	65 (62.5)	1 (0.96)	
Occupation					
Employed [*n* (%)]	1 (1.92)	21 (40.38)	30 (57.69)	0	0.93²
Unemployed [*n* (%)]	2 (2.04)	36 (36.73)	59 (60.2)	1 (1.02)	
Education					
Illiterate [*n* (%)]	0	7 (29.16)	17 (70.83	0	0.9²
Primary school [*n* (%)]	2 (3.5)	25 (43.85)	29 (50.87)	1 (1.75)	
High school [*n* (%)]	1 (1.88)	19 (33.84)	33 (62.66)	0	
Bachelor's D. [*n* (%)]	0	5 (45.45)	6 (54.54)	0	
Master's D. [*n* (%)]	0	1 (25)	3 (75)	0	
Ph.D D. [*n* (%)]	0	0	1 (100)	0	
Marital status					
Single [*n* (%)]	0	3 (18.75)	13 (81.25)	0	
Married [*n* (%)]	3 (2.23)	54 (40.29)	76 (56.71)	1 (0.74)	
Cancer					
Hematologic [*n* (%)]	0	10 (38.46)	16 (61.53)	0	<
Non‐hematologic [*n* (%)]	3 (2.41)	47 (37.9)	73 (58.87)	1 (0.8)	0.99²
Surgery [*n* (%)]	2 (2.27)	32 (36.36)	53 (60.22)	1 (1.13)	0.93²
Chemotherapy [*n* (%)]	3 (2.18)	50 (36.49)	83 (60.58)	1 (0.72)	0.57²
Radiotherapy [*n* (%)]	1 (2.7)	10 (27.02)	26 (70.27)	0	0.33²
Patient condition					
Under treatment [*n* (%)]	1 (1.2)	36 (43.37)	46 (55.42)	0	0.26²
Palliative therapy [*n* (%)]	2 (2.98)	21 (31.34)	43 (64.17)	1 (1.49)	
Treatment methods					
Chemotherapy + Radiotherapy [*n* (%)]	1 (3.12)	9 (28.12)	22 (68.75)	0	0.46²
Chemotherapy + surgery [*n* (%)]	2 (2.5)	28 (35)	49 (61.25)	1 (1.25)	0.79²
Radiotherapy + surgery [*n* (%)]	0	8 (28.57)	20 (71.42)	0	0.50²
Radiotherapy + surgery + chemotherapy [*n* (%)]	0	7 (30.43)	16 (69.56)	0	0.74²

*Note:* Data are presented as mean ± standard deviation (SD) and range for continuous variables, and *n* (%) for categorical variables. P‐values were calculated using ANOVA or Fisher's exact test. Significance set at *p* < 0.05.

^¹^ Analyzed by the Analysis of Variance (ANOVA) test.

^²^ Analyzed by Fisher's exact test.

## 4 Discussion

In the present study, breast cancer was the most prevalent type, and neither type comprised the majority. All of the participants in the current study were cancer patients; some were in advanced stages and suffered from cachexia. circadian rhythm distribution in this study was normal. In this study, we combined data from all cancer types to provide a general view of circadian rhythm distribution.

Several cancers are associated with circadian clock dysfunction, highlighting the connection between circadian rhythm dysregulation and oncogenesis [29]. Epidemiological studies have linked cancers to night shift work and light pollution that disrupt chronotype [2, 30]. In mice, dysregulated circadian gene expression may cause lymphoma, osteosarcoma, and hepatocellular carcinoma (HCC), according to Kettner et al. [31].

Jiang et al. reported that circadian gene disruption is associated with the onset of HCC [11]. Methylation of single nucleotide polymorphisms (SNPs) or clock gene promoters is a known molecular mechanism [30]. Consideration of this issue could lead to cancer prevention and treatment in the future.

In our study, there was no significant correlation between chronotype and gender (*p* = 0.629), age (*p* = 0.135), marital status (*p* = 0.263), occupation (*p* = 0.931), or education level (*p* = 0.899). Definitive and moderate morning types exhibited higher age means than neither and moderate evening types. This finding is consistent with the findings of Montaruli et al. who observed that older adults are typically morning types, possibly due to age‐related sleep shortness, whereas younger adults are commonly evening types [32]. Furthermore, there is no correlation between age and gender, and circadian rhythm [32, 33, 34]. Some studies suggest that there may be gender differences in circadian types due to housework, grooming, and breakfast preparation. While circadian rhythms exist across all individuals, research indicates that the timing and robustness of these rhythms can vary based on age and gender. Women often show a phase advance in circadian markers such as melatonin secretion and core body temperature, while older adults tend to shift toward a morning‐type preference regardless of prior chronotype. These physiological variations may contribute to differences in chronotype distribution observed in our cancer patient population [35].

In a cohort study conducted by Ramin et al. the average age and BMI of the participants were 59.2 y and 27.5 kg/m^2^, respectively, and the most prevalent chronotype was definitely morning type, while neither type was the least prevalent [36].

Neither type increased the risk of breast cancer among participants, but the difference was not statistically significant [36]. This study which examined 72,517 patients, reported a significant association between breast cancer patients and the “neither” chronotype group. This discrepancy may stem from differences in the study populations, as their cohort included only breast cancer patients, whereas our study analyzed multiple cancer types. Additionally, differences in the distribution of chronotype groups or sample size may have influenced the results.

Our study's mean age and BMI were 49 and 25.09 kg/m^2^, respectively. All of the participants in the current study were cancer patients; some were in advanced stages and suffered from cachexia, which could explain the differences in BMIs. Bhar et al. found that evening types have a higher BMI, FBS, and HbA1c, resulting in less physical activity, unhealthy eating habits, and sleep disturbances that lead to T2DM [33, 37, 38]. Although the overall mean BMI was in the overweight range in our study, it is important to note that BMI varied widely among participants. Some patients, particularly those with advanced disease or under palliative care, exhibited weight loss that resulted in lower BMI values. This variability may reflect the complex interaction between cancer progression, treatment type, and nutritional status.

In contrast to our study, the mean BMI is higher for morning types, but no significant difference was

observed between circadian types (*p* = 0.317). The participants lacked comorbidities such as diabetes, and we did not evaluate their physical activity and dietary habits; as previously mentioned, some patients were in the cachectic phase.

It is pertinent to note that Circadian rhythms are closely linked to sleep‐wake hormones such as melatonin, cortisol, and prolactin, which in turn influence immune function. Cancer‐related immune markers, such as pro‐inflammatory cytokines, may follow circadian rhythms, and disruptions in these rhythms could impact tumor progression and metastasis [39, 40]. Furthermore, recent studies suggest that tumor‐derived RNA and DNA is released into the bloodstream in a rhythmic manner, potentially offering insights into circadian influence on cancer dynamics and response to the treatment [41, 42]. Chronotype was not significantly associated with duration as a cancer patient (*p* = 0.855) or hospital admission (*p* = 0.250). The neither and moderately evening types developed cancer sooner, were hospitalized more frequently, experienced fatigue and weakness, and were in advanced stages; consequently, these groups exhibit a lower BMI. The present study observed normal distribution for chronotype, and the mean MEQ score was 56.6 ± 6.34 [40]. Neither type was the most chronotype, with the moderately evening type being the least. No evening‐only types were detected. In a case‐control study conducted by Di Somma et al. the mean MEQ score of craniopharyngioma patients was 47.8 ± 12.6 [34].

Most participants were morning types, and the minority were evening types [34]. Definitive and moderate morning types were considered one group, and evening types were considered a second [34]. Based on the results, females were predominantly evening and intermediate types, while males were primarily morning types. Different MEQ means and scores may result from the sample size, study design, and various types of cancer. In line with our findings, Kanagarajan et al. demonstrated that the MEQ distribution in bipolar patients aged 25–66 was normal, the mean score was 49.2 ± 10.4 [24], neither type was the most chronotype, and circadian rhythm was not significantly associated with age and gender [43]. The correlation between circadian rhythm and surgery (*p* = 0.933), chemotherapy (*p* = 0.565), and radiotherapy (*p* = 0.326) were not statistically significant Most patients who received chemotherapy and radiotherapy were neither morning nor evening types, whereas morning types had more chemotherapy sessions. Multiple studies have suggested that a higher MEQ score for morning types is associated with fewer chemotherapy‐related side effects, such as nausea and vomiting [32]. Cancer patients were the subject of a case‐control study by Sultan et al. [44]. The participants' mean age was 46.67 ± 12.51 y [44]. Similar to our research, the majority and minority circadian rhythms were neither morning nor evening. The MEQ score was negatively correlated with chemotherapy‐induced nausea, vomiting, and diarrhea [44]. Unfortunately, we did not assess the side effects of chemotherapy or radiotherapy. Hematologic and non‐hematologic cancers (*p* = 0.999), palliative therapy or treatment (*p* = 0.262), treatment method combinations such as chemotherapy + radiotherapy (*p* = 0.457), chemotherapy + surgery (*p* = 0.794), radiotherapy + surgery (*p* = 0.500), and chemotherapy + radiotherapy + surgery (*p* = 0.738) were not significantly correlated to chronotype. The majority of participants in these groups fell into neither category. The predominance of “neither type” chronotype in cancer patients may suggest a disruption in the body's natural circadian rhythm, potentially contributing to the dysregulation of biological processes such as cell division, immune function, and response to treatment. Further studies are needed to explore the clinical relevance of this chronotype in cancer progression and treatment outcomes [45].

Some research indicates that chrono chemotherapy and chrono radiotherapy may improve cancer patients' survival and response rate [18]. Consequently, chrono modulated chemotherapy or radiotherapy sessions compatible with circadian rhythm may be advantageous to the treatment process [18]. Although a disorganized circadian rhythm may result in carcinogenesis, cancer treatments may alter the circadian type. Comparing the chronotype of various cancers requires additional research. Chrono‐modulated cancer therapies are primarily designed to minimize treatment‐related side effects by aligning drug administration with the circadian rhythms of healthy tissues. However, current evidence suggests that tumor cells often operate on an irregular or even independent circadian cycle, making them less responsive to such timing strategies. Therefore, rather than enhancing tumor control directly, the key benefit of chronotherapy lies in protecting normal cells and improving patients' tolerance to treatment. These insights emphasize the need for future studies focused on personalized chronotherapeutic planning [46, 47].

## 5 Study Limitations

Central circadian rhythms, primarily regulated by the SCN, are entrained by light exposure, whereas peripheral clocks are strongly influenced by other factors such as food intake and exercise [48, 49]. Considering food intake as a major driver of peripheral rhythms, future studies should include a detailed dietary questionnaire to explore its potential association with chronotype in cancer patients.

Assessing the circadian rhythm at the time of diagnosis and the lack of longitudinal data collection at specific time points and then comparing it during treatment and follow‐up, not having a control group, having a small sample size, resulted in limited statistical power. In this study, we combined data from all cancer types to provide a general view of circadian rhythm distribution. However, we acknowledge that different cancer types may affect circadian rhythm independently, and future studies should consider stratifying data by cancer type to assess potential differences. As MEQ was administered only once regardless of treatment phase, we acknowledge this may not reflect dynamic changes in circadian rhythm caused by treatment. Also it is important to note that due to the latency of cancer development, chronotype assessed during active disease may not reflect the pre‐morbid circadian pattern. Factors such as treatment, aging, or lifestyle changes may influence current chronotype.

## 6 Conclusion

In conclusion, this study found a predominance of the “neither type” chronotype among cancer patients, which may suggest a disruption in their circadian regulation. Given the growing body of evidence linking circadian rhythm disturbances to cancer development, progression, and treatment response, these findings highlight the importance of assessing biological timing and targeting the clock genes in oncological managements to improve prognosis. Although the Morningness‐Eveningness Questionnaire provides a snapshot of chronotype, further research is needed to explore dynamic circadian patterns throughout the cancer care. Ultimately, integrating circadian profiling into routine clinical assessments may help for more personalized and time‐sensitive approaches to treatment, such as chronotherapy, with the aim of improving tolerability and potentially enhancing therapeutic outcomes.

## Author Contributions

**Ghazal Daftari:** data curation, writing – original draft, formal analysis, investigation. **Mahsa Abbaszadeh:** conceptualization, writing – review and editing, supervision. **Sahar Karimpour Reyhan:** supervision, writing – review and editing, investigation. **Nasim Khajavirad:** conceptualization, methodology, supervision, validation, investigation.

## Ethics Statement

Informed written consent was obtained from participants. All procedures performed in studies involving human participants adhered to the ethical standards of the institutional and/or national research committee and with the 1964 Helsinki declaration and its later amendments or comparable ethical standards. A copy of the written consent is available for review by the Editor‐in‐Chief of this journal.

## Conflicts of Interest

This study received no external funding. The authors declare that no funding source had any role in the design, data collection, analysis, interpretation, writing, or decision to publish this study. The lead author, Dr. Ghazal Daftari, affirms that this manuscript is an honest, accurate, and transparent account of the study being reported; that no important aspects of the study have been omitted; and that any discrepancies from the study as planned have been explained.

## Transparency Statement

The lead author Nasim Khajavirad affirms that this manuscript is an honest, accurate, and transparent account of the study being reported; that no important aspects of the study have been omitted; and that any discrepancies from the study as planned (and, if relevant, registered) have been explained.

## Supporting information

Appendix A.

## Data Availability

The data that support the findings of this study are available on request from the corresponding author. The data are not publicly available due to privacy or ethical restrictions.
