# Supplementary material for: Cancer Patients Circadian Rhythm Assessment Based on Morningness‐Eveningness Preference: A Cross‐Sectional Study
Source: Health Sci Rep. 2025 Sep 3;8(9):e71210. doi: 10.1002/hsr2.71210 (PMC12405965; doi:10.1002/hsr2.71210)
Supplement: Supplementary file 1 — Appendix A. [file HSR2-8-e71210-s001.docx]

**Appendix A. Clinical and Demographic Questionnaire**

This questionnaire was used to collect baseline demographic and clinical data from cancer patients enrolled in the study. It was reviewed and approved by subject-matter experts for content validity.

**English Version**

Please complete the following information. All responses are confidential and will be used for research purposes only.

Section 1: Demographic Information
1. Gender: ☐ Male ☐ Female ☐ Other
2. Age: ______ years
3. Height: ______ cm
4. Weight: ______ kg
5. Marital Status: ☐ Single ☐ Married ☐ Divorced ☐ Widowed
6. Educational Level:
 ☐ Illiterate ☐ Primary School ☐ High School ☐ Bachelor’s Degree ☐ Master’s Degree ☐ Ph.D.
7. Occupation: ☐ Employed ☐ Unemployed ☐ Retired ☐ Student ☐ Other: ____________

Section 2: Clinical Information
8. Type of Cancer: ________________________
9. Date of Cancer Diagnosis (Month/Year): ____________
10. Duration of Illness (in months): ________
11. Number of Hospitalizations related to cancer: ________
12. History of Treatments (check all that apply):
 ☐ Chemotherapy ☐ Radiotherapy ☐ Surgery ☐ None yet
13. Number of chemotherapy sessions (if applicable): ________
14. Number of radiotherapy sessions (if applicable): ________
15. Surgery (if applicable): Date and Type: ____________________________
